# Effects of the cryopreservation process on dog sperm integrity

**DOI:** 10.21451/1984-3143-AR2019-0081

**Published:** 2020-03-24

**Authors:** Carmen Cecilia Sicherle, Fabiana Ferreira de Souza, Camila de Paula Freitas-Dell’Aqua, Gabriele Barros Mothé, Carlos Roberto Padovani, Frederico Ozanam Papa, Maria Denise Lopes

**Affiliations:** 1 Departamento de Cirurgia Veterinária e Reprodução Animal, Universidade Estadual Paulista “Julio de Mesquita Filho”, Botucatu, SP, Brasil; 2 Departamento de Bioestatística, Instituto de Biociências, Universidade Estadual Paulista “Julio de Mesquita Filho”, Botucatu, SP, Brasil

**Keywords:** dog, spermatozoa, cryopreservation, sperm quality, cell integrity

## Abstract

Sperm cryopreservation has become an indispensable tool in reproductive biology. However, frozen/thawed semen has a short lifespan due to loss of sperm cell integrity. To better understand which sperm cell structures are compromised by the cryopreservation process and apoptosis markers, the sperm of five healthy mature dogs was analyzed in this study. Analysis was performed after collection, cooling, and thawing via computer assisted sperm analyzer (CASA) and evaluation of membrane fluidity and permeability, phosphatidylserine translocation (Annexin V), membrane integrity, mitochondrial membrane potential, membrane lipid peroxidation (LPO) and activity of the apoptotic markers caspases 3 and 7 by flow cytometry. Cryopreservation decreased total and progressive motility and the percentage of rapid sperm (*P <* 0.01). Damage to sperm cells was confirmed by Annexin V (*P <* 0.01), indicating that capacitation-like changes were induced by the cryopreservation procedures. An increase in sperm membrane fluidity was also noted in frozen/thawed samples (*P <* 0.01). Plasma and acrosomal cell membranes were affected (*P <* 0.01), with decreases in the subpopulation displaying high membrane potential (*P <* 0.01). Membrane LPO was increased in thawed sperm compared to cooled sperm (*P <* 0.05) but was not different from that in fresh sperm. No differences were observed in caspase 3 and 7 activity after cooling, freezing, or thawing. In conclusion, total and progressive motility, plasma membrane integrity and mitochondrial membrane potential suffered from the deleterious effects caused by cryopreservation, unlike the activity of caspases that remained stable during the freezing process.

## Introduction

Cryopreservation of semen offers practical advantages because there is no dependence on time or distance, and it can indefinitely preserve the genetics of higher animals. However, frozen/thawed dog semen has a short lifespan due to loss of sperm cell integrity during the processes of cooling, freezing and thawing (Alhaider and Watson, 2009). Moreover, the fact that the bitch ovulate immature oocytes, which take 2 to 3 days to be read for fertilization, creates difficulties in determining the optimum time for insemination, making it even more important long functional life of the sperm after thawing (Peña et al., 1999; Alhaider and Watson, 2009; Farstad, 2012). Regardless, the viability of sperm when analyzed by motility is usually greater than the actual fertilization capacity due to changes that occur in cell membranes during the cryopreservation process (Maxwell and Watson, 1996). Overall, the decreased lifespan of thawed sperm cells is due to changes in the cell membrane that are similar to those of capacitation or are attributed to a cellular degenerative process (Sokolowska et al., 2009; Ortega-Ferrusola et al., 2017).

Changes in the fluidity of the sperm membrane associated with intracellular accumulation of calcium and increases in tyrosine phosphorylation characterize sperm cell capacitation (Niżański et al., 2012; Niżański et al., 2016; Ortega-Ferrusola et al., 2017). The degenerative processes of sperm can be classified as necrosis or apoptosis, whereby in the latter, the cell plays an active role in its own dissolution through ultrastructural and biochemical modifications coordinated by genetic and molecular induction (Câmara and Guerra, 2008). Moreover, in addition to other factors, apoptosis is associated with activation of caspases, loss of mitochondrial membrane potential and transposition of phosphatidylserine (PS) to the outer leaflet of the sperm cell membrane (Hossain et al., 2011; Petrunkina and Harrison, 2013; Ortega-Ferrusola et al., 2017).

Many different probes have been used to assess cell structures for examining the integrity and possible viability of semen samples. For example, the fluorescent probe merocyanine 540 (M540) detects changes in membrane lipid arrangement, an initial modification in capacitation: as fluidity increases, more stain can bind to the membrane, acting as a marker of membrane destabilization (Harrison et al., 1996; Rodríguez-Martínez, 2003; Niżański et al., 2012; Steckler et al., 2015). The dye Yo-Pro-1 (YP) penetrates the cell upon plasma membrane destabilization and an increase in the permeability of pannexin-gated channels, even before complete loss of membrane integrity occurs (Peña et al., 2005). When the plasma membrane ruptures, PS moves from the inside to the outside of the membrane, identifying primary damage (Kim et al., 2010b), and the calcium-dependent probe Annexin V is used to detect PS externalization (Hossain et al., 2011; Petrunkina and Harrison, 2011). In general, loss of sperm mitochondrial membrane integrity is an alteration that marks one of the pathways of apoptotic-like changes observed after cryopreservation (Martin et al., 2004). To date, different probes have been used to evaluate mitochondrial membrane potential (MMP), such as MitoTracker Deep Red, Red, Orange, and Green and JC-1 (Gadella and Harrison, 2002; Sousa et al., 2011; Niżański et al., 2012;).

Plasma membrane lipid peroxidation (LPO) is a physiological event in which radicals from oxygen metabolism (reactive oxygen species [ROS]) react with membrane lipids in an oxidative process that prepares spermatozoa for fertilization. However, high levels of ROS cause destruction of the lipid matrix structure, leading to a loss of membrane integrity. In frozen/thawed dog semen, LPO increases the PS translocation index, intracellular hydrogen peroxide levels and DNA fragmentation compared to the levels of fresh semen (Kim et al., 2010b). Indeed, LPO may be the cause of 20 to 40% of human infertility (Agarwal et al., 2004). In one study, the efficiency of staining to mark cells that have undergone membrane LPO was confirmed in human sperm after an induced reaction with iron (Aitken et al., 2007).

As cryopreservation causes different levels of damage to spermatozoa, a better understanding of cryodamage is necessary to achieve greater fertility results in canines. Thus, to investigate which sperm cell structures are compromised by the cryopreservation process and apoptosis markers, the objective of the present study was to evaluate the effects of cryopreservation on sperm cell membrane integrity, mitochondrial membrane potential, membrane LPO and caspase activity.

## Methods

### Animals

Five adult healthy dogs of different breeds with sperm motility greater than 70% were used in this study. The ages of the animals ranged from 3 to 5 years of age and weighs from 25 to 30 kg. The ethics committee of the university reviewed and approved the experimental design (number 133/2014-CEUA).

### Reagents

All reagents used were obtained from Sigma-Aldrich (St.Louis, USA) and Merck SA (St.Louis, USA), unless otherwise indicated.

### Semen collection and processing

Semen was collected into prewarmed graduated tubes by digital manipulation. No teaser female was used. Four samples from each dog were collected on a weekly basis (20 ejaculates). After collection, an aliquot of the sperm sample was evaluated for sperm motility, concentration, and morphology, as well as flow cytometry analyses. The semen was centrifuged for 10 min at 800 × *g*. The seminal plasma was then removed, and the sperm pellet was resuspended in one step in Tris-egg yolk extender at room temperature (20 °C) (200 mM Tris, 67 mM citric acid, 44.4 mM D-fructose, 20% egg yolk, 8% glycerol, Orvus WA paste [Procter & Gamble, Cincinnati, OH, USA], 0.02% amikacin sulfate and distilled water qsp) (Morton, 1998) as modified in another study (Chirinéa et al., 2006), resulting in a concentration of 80 × 10^6^ sperm per mL. After dilution, the semen was loaded into 0.5-mL French straws and cooled to 5 °C for 1 hour (equilibration time). The samples were frozen horizontally in racks by placing at 6 cm above the surface of liquid N_2_ in a closed Styrofoam box for 20 min and then immersing directly into liquid N_2_. At the time of analysis, the straws were thawed in a water bath at 46 °C for 20 s (Chirinéa et al., 2006). All experimental analyses were performed using fresh, cooled and thawed semen.

### Motility analysis

Motility of fresh, cooled and thawed sperm samples was measured using a CASA system (HTMA-IVOS 10, IMV, Beverly, MA, USA) according to a previously described protocol (Verstegen et al., 2002). The percentages of total motility (TM), progressive motility (PM) and rapid cells (RAP) were evaluated.

### Flow cytometry

Flow cytometry analyses were carried out using an LSR Fortessa (BD Biosciences) equipped with three laser excitation sources (405 nm and 50 mW; 488 nm and 50 mW; 640 nm and 40 mW) that were quality-controlled on a daily basis using CS&T beads and FACS DiVA software (BD Biosciences). The filter configurations for the PMTs measuring the fluorescence emission of the applied fluorochromes were 530/30 nm (FITC; for Annexin V, PSA, JC-1, and BODIPY-C11), 694/50 nm (PI; for PI, JC-1 and BODIPY-C11), and 450/50 nm (Hoescht 33342). Data were plotted using biexponential plots with axes < 0 to ensure that all data were visible and properly compensated for.

Samples were diluted in TALP-PVA as described by Parrish et al. (1988), with modification (100 mM NaCl, 3.1 mM KCl, 25.0 mM NaHCO_3_, 0.3 mM NaH_2_PO_4_, 21.6 mM sodium DL-lactate 60%, 2.0 mM CaCl_2_, 0.4 mM MgCl_2_, 10.0 mM HEPES free acid, 1.0 mM sodium pyruvate, 1.0 mg/mL polyvinyl alcohol [PVA] and 25 µg/mL gentamicin sulfate) to achieve a sperm concentration of 5 × 10^6^ sperm/mL. Hoescht 33342 (7 µM) was included to distinguish sperm cells from other artifacts.

Sperm plasma and acrosomal membrane integrity was assessed using propidium iodide (1.5 µM; P4170, Sigma-Aldrich, St. Louis, USA), FITC-PSA (0.2 ng/mL; L-0770, Sigma-Aldrich) and Hoechst 33342 (7 µM; 14533, Sigma-Aldrich), as previously described (Mothé et al., 2018); samples were incubated at 37 °C for 15 min in the dark. To evaluate phosphatidylserine membrane translocation, an Annexin V-FITC Kit I for apoptosis estimation was used (550475; BD Bioscience Pharmingen) with PI and Hoechst according to the manufacturer’s instructions; the samples were incubated at 37 °C for 15 min in the dark. To evaluate membrane fluidity and permeability, merocyanine (2.7 µM; M24571) and Yo-Pro 1 (25 nM; Y3603, Molecular Probes, Inc., Eugene, Oregon) were utilized according to methods described previously (Steckler et al., 2015).

A lipid peroxidation assay was performed using a BODIPY-C11 probe (D-3861; Molecular Probes Inc.) with Hoechst 33342 and PI according to methods described previously (Guasti et al., 2012). To 499.5 µL of diluted semen in TALP-PVA at 1 × 10^6^ sperm/mL, 1 μM BODIPY-C11, 7 μM Hoechst and 1.5 μM PI were added, and the sample was incubated for 20 min at 37 °C. After incubation, the sample was centrifuged at 300 × *g* for 5 min (twice) and reconstituted to 500 μL. For positive control it was used 80 μM of ferrous sulfate (FeSO_4_) and 5 μM cumene hydroperoxide (C_6_H_5_C(CH_3_)_2_OOH) (Sigma-Aldrich, St. Louis, USA).

For caspase activity evaluation, a caspase 3/7-FITC kit (C10427, Molecular Probes Inc.) with PI and Hoechst was used according to the manufacturer's recommendations, and the samples were incubated at 37 °C for 25 min in the dark. To assess mitochondrial membrane potential, JC-1 (153 µM; T3168, Molecular Probes Inc.) was employed according to methods described previously (Mothé et al., 2018), with incubation for 10 min at 37 °C.

### Statistical analysis

The results are presented as means ± standard error. Statistical analysis of the data was performed using the Kolmogorov-Smirnov test for normality followed by Bonferroni multiple comparisons (Johnson and Wichern, 1988). Results were considered significant at the *P <* 0.01 and *P <* 0.05 levels.

## Results

**Figure 1:**
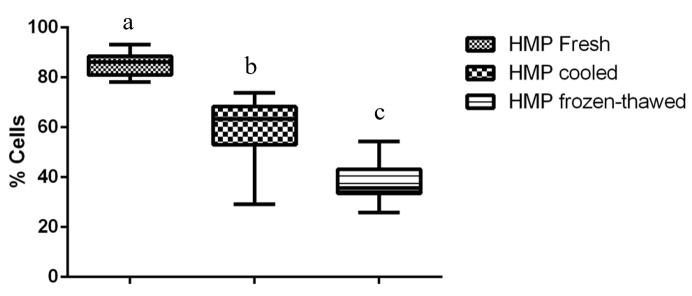
Percentage of cells displaying high membrane potential (HMP) on fresh, cooled and frozen-thawed dog semen. Values with different superscripts differ significantly: *P < 0.01*.

**Figure 2:**
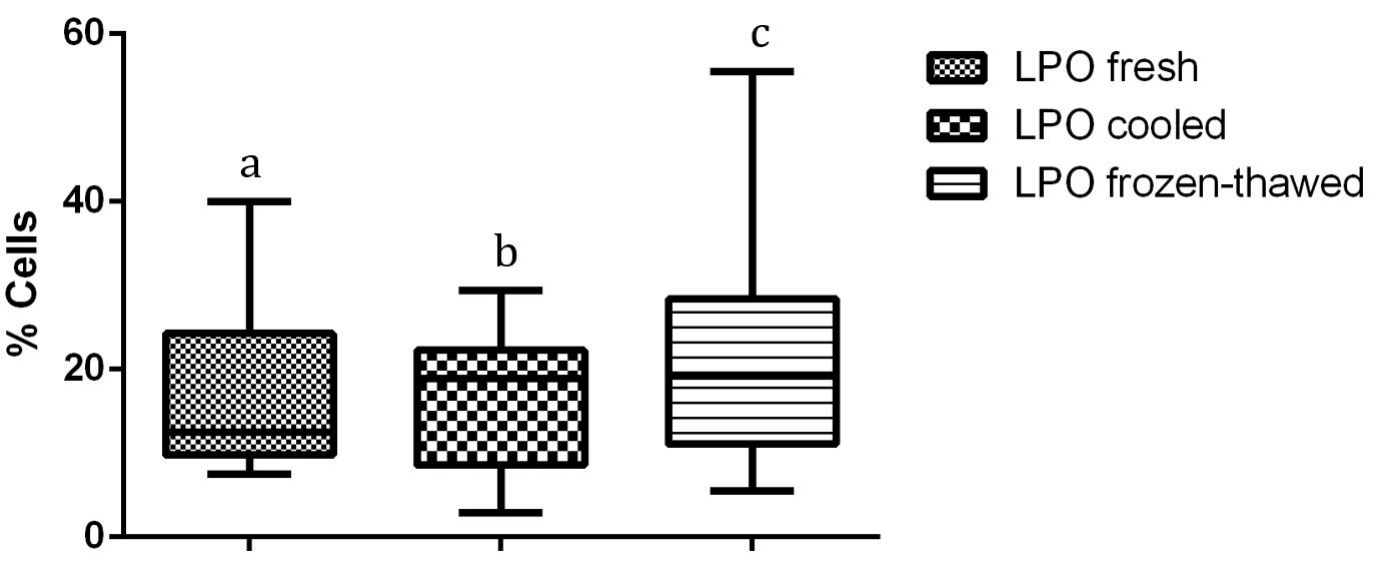
Percentage of cells presenting membrane lipoperoxidation on fresh, cooled and frozen-thawed dog semen. Values with different superscripts differ significantly: *P < 0.05*.

**Figure 3:**
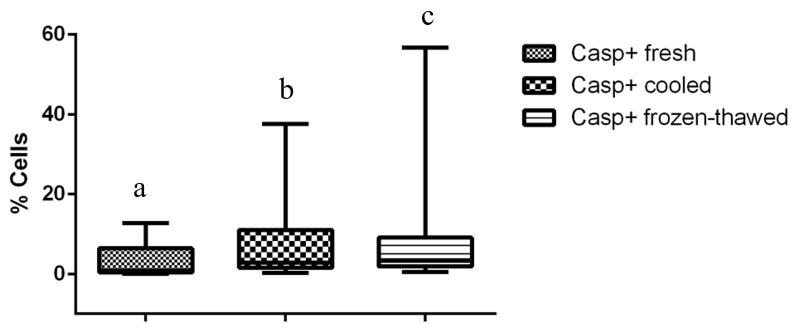
Percentage of cells with caspase 3/7 activity on fresh, cooled and frozen-thawed dog semen. Values with different superscripts differ significantly: *P < 0.01*.

Compared to fresh and cooled semen, cryopreservation treatment of sperm led to a significant decrease in total and progressive motility and in the percentage of cells displaying rapid movements (*P <* 0.01) (Table 1). Flow cytometry data revealed the cryodamage to sperm cells, whereby massive movement of PS to the outer leaflet membrane occurred after thawing, decreasing the number of intact cells, as confirmed by Annexin V staining (*P <* 0.01). Additionally, an evident subpopulation of fresh sperm exhibiting PS translocation that was not modified by cooling or cryopreservation processes was observed (*P <* 0.01) (Table 2). An increase in sperm membrane fluidity was also noted. In the subpopulation that was not permeable to YP (*P <* 0.01) and in samples showing an increase in YP uptake, there was a common M540-fluorescent subpopulation among frozen/thawed samples, indicating an increase in cell membrane permeability and compromised membranes after cryopreservation (*P <* 0.01). The acrosomal cell membrane was also affected, as assessed by FITC-PSA/PI (*P <* 0.01), and a decrease in intact cells was noted. Similarly, the mitochondrial membrane was affected by cryopreservation, with a reduction in the subpopulation displaying a high membrane potential being observed after cooling and after thawing (*P <* 0.01) (Figure 1). Moreover, an increase in membrane LPO was observed for thawed sperm compared to that for cooled sperm, but the membrane LPO of thawed sperm was not different from that of fresh sperm (*P <* 0.05) (Figure 2). Conversely, no differences were observed with regard to caspase 3/7 activity (Figure 3).

**Table 1:** Means ± standard error of parameters analyzed in fresh, cooled and frozen/thawed dog semen, as expressed as percentages of total sperm cells. Total motility (TM), Progressive motility (PM), Rapid sperm (RAP).

**Parameter**	**Moment**		
TM	87.00 ± 1.24^a^	88.15 ± 1.38^a^	72.55 ± 6.26^b^
PM	69.95 ± 1.28 ^a^	71.75 ± 1.91^a^	56.30 ± 6.00^b^
RAP	83.15 ± 1.94^a^	81.55 ± 1.53^a^	64.30 ± 7.68^b^

Within a row, values with different superscripts differ significantly; *P <* 0.01 (n = 20 ejaculates).

**Table 2:** Means ± standard error of parameters analyzed in fresh, cooled and frozen/thawed dog semen, as expressed as percentages of total sperm cells. Membrane Fluidity and Permeability (YP/M540), Phosphatidylserine Translocation (AN/PI) and Membrane Integrity (FITC/PSA/PI).

**Parameter**	**Moment**		
YP-/M540-	78.29 ± 6.22^a^	75.76 ± 6.60^a^	23.02 ± 9.12b
YP-/M540+	18.33 ± 5.54^a^	22.55 ± 6.49^a^	71.78 ± 9.85^b^
AN-/PI-	79.90 ± 7.95^a^	63.50 ± 4.66^b^	27.19 ± 9.22^c^
AN+/PI-	2.23 ± 2.06	6.88 ± 2.12	8.25 ± 10.16
FITC-PSA-/PI-	85.71 ± 6.22^a^	70.37 ± 6.43^a^	30.87 ± 9.90^b^

Within a row, values with different superscripts differ significantly; *P <* 0.01 (n = 20 ejaculates). YP-/M540-, FITC-PSA-/PI-: cells displaying intact plasma and acrosomal membranes; YP-/M540+: cells with no permeability to YO PRO but increased fluidity (permeable to Merocyanine 540); AN-/PI-: no PS translocation; AN+/PI-: cells with PS translocation but not permeable to propidium iodide.

## Discussion

In the present study, we evaluated and compared the effects induced by the cooling and freezing steps of cryopreservation that may impair cell viability in canine sperm. Sperm cells need to exhibit forward motility and be morphologically capable to penetrate an oocyte. Despite a significant decline in motility parameters after cooling and thawing in this study, the percentage of sperm demonstrating progressive motility was greater than 50%; which is considered sufficient to be used in AI with frozen semen in dogs.

After a freeze/thaw cycle sperm lose the capacity to adhere to the epithelial cells of the oviduct, and this probably occurs due to an increase in the number of sperm having undergone capacitation-like changes and acrosome reactions (Burgess et al., 2012). Sperm membrane integrity is in addition to motility, also extremely important for successful fertilization (Kim et al., 2010a; Rota et al., 2010; Mason and Rous, 2014). The changes in temperature that occur during cryopreservation are the main causes of alterations to the sperm membrane, such as the increase in permeability and fluidity, what can be revealed by YP and M540 analysis. Although M540 attaches to the sperm membrane and fluoresces according to the degree of membrane disorganization, YP penetrates cells with compromised plasma membranes.

In our study, we observed a subpopulation with decreased membrane fluidity and permeability (M540+ e YP) among the cooled and thawed samples; however, a subpopulation with decreased membrane fluidity (M540+) but without YP permeability was also present, confirming that dog sperm cells undergo capacitation due to cryopreservation. These subtle changes, as detected by the M540 and YP, alter the membrane lipid architecture and reduce the enzymatic activity necessary for the extrusion of calcium ions, which leads to premature membrane fusion and shortens the timing of capacitation (Niżański et al., 2012). As YP can penetrate the plasma membrane in the early stages of damage, it is a useful indicator for predicting cell death in dog sperm, and the combination of YP and M540 is thus a sensitive indicator of cell status (Steckler et al., 2015).

In the present study, we found a significant decrease in the number of intact cells in cooled and frozen/thawed semen samples, showing that cooling and freezing/thawing processes cause PS translocation in dog spermatozoa, as previously reported (Kim et al., 2010b), and confirming that capacitation-like changes are induced by cryopreservation procedures.

In contrast, the sperm population that already displayed PS translocation in fresh semen was not affected by cooling or cryopreservation, as previously shown in both dogs and horses and in the present study. This fact emphasizes the heterogeneous nature of sperm cells and the fact that some populations exit the epididymis destined to die prematurely or to become targets of the female reproductive tract immune system (Kim et al., 2010b; Ortega-Ferrusola et al., 2017). Annexin V, which marks PS translocation, can detect early stages of membrane damage, which is useful for assessing the responses of sperm cells to stressful conditions such as cooling and freezing processes (Petrunkina and Harrison, 2011) and serve as an additional tool for determining which individuals are “good” or “bad” freezers, as individual resistance to sperm cryopreservation has been reported to occur in the males of many species (Nunez-Martínez et al., 2006; Ortega-Ferrusola et al., 2009; Ramón et al., 2013). PS translocation caused by cryopreservation has been observed in frozen/thawed semen samples from humans, boars, bulls, horses and dogs (Duru et al., 2001; Anzar et al., 2002; Peña et al., 2003; Kim et al., 2010b; Ortega-Ferrusola et al., 2017), indicating that the freezing process decreases the integrity of sperm cells, which has a definite impact on their viability and fertilization capacity post-thaw.

Cryopreservation also decreased the mitochondrial membrane potential of sperm after cooling and freezing/thawing. Such loss of mitochondrial membrane integrity by any means causes mitochondrial pore formation, leading to decreased mitochondrial activity and the liberation of proapoptotic factors into the cytoplasm, which decreases the lifespan of a sperm cell and begins a degradation phase (Niżański et al., 2012; Niżański et al., 2016).

Low MMP is not always related to poor sperm motility. If the energy necessary for motility is generated from oxidative phosphorylation due to the enzymatic complex present on the internal mitochondrial membrane, a proportional effect on sperm motility would be observed. However, this phenomenon was not observed in the present study: a massive effect on the sperm subpopulation displaying HMP, but not on motility parameters, was found. A positive correlation between static cells and high MMP has been reported for canine (Volpe et al., 2009; Lucio et al., 2016) and human (Donnelly et al., 2000) semen, which suggests that mitochondrial activity is not the only source of energy for sperm motility. Indeed, glycolysis in the midpiece is more likely than oxidative phosphorylation to provide the energy for sperm motility (Turner, 2003; Petrunkina and Harrison, 2011). Although a decrease in MMP is one of the factors that initiates apoptosis, the involvement of mitochondria in apoptosis, is characterized by activation of cysteine proteases known as caspases (Donnelly et al., 2000; Câmara and Guerra, 2008). In the present study, differences in the activities of caspase 3 and 7, known as effector caspases, were not observed in fresh, cooled or thawed semen. It is not yet fully clear whether apoptosis actually occurs in ejaculated sperm or only in those species where a cytoplasm residue is present, such as in humans and stallions (Hossain et al., 2011). Although apoptosis-like changes, such as PS translocation and decreased mitochondrial potential, were found after cryopreservation in the present study, they appear to be related to cryoinjury rather than apoptosis.

A significant difference in LPO was observed between cooled and frozen/thawed samples. This result is probably due to the stress of freezing procedure, including the manipulation, leading to an increase in ROS, which is an inducer of LPO and therefore an increase in ROS is another factor that indicates that a cell has suffered a stress (Donnelly et al., 2000; Câmara and Guerra, 2008; Kim et al., 2010a; Lucio et al., 2016; Ortega-Ferrusola et al., 2017).

## Conclusion

In conclusion, total and progressive motility, plasma membrane integrity and mitochondrial membrane potential suffered from the deleterious effects caused by cryopreservation, unlike the activity of caspases that remained stable during the freezing process.
